# ^1^H, ^13^C, and ^15^N backbone and methyl group resonance assignments of ricin toxin A subunit

**DOI:** 10.1007/s12104-024-10172-8

**Published:** 2024-04-20

**Authors:** Shibani Bhattacharya, Tassadite Dahmane, Michael J. Goger, Michael J. Rudolph, Nilgun E. Tumer

**Affiliations:** 1 New York Structural Biology Center, 89 Convent Avenue, New York, NY 10027 USA; 2 Department of Plant Biology and Pathology, School of Environmental and Biological Sciences, Rutgers University, 59 Dudley Road, New Brunswick, NJ 08901-8520 USA

**Keywords:** Allostery, Chemical shift mapping, Nuclear magnetic resonance spectroscopy, P-stalk protein, Ribosome, Ribosome inactivating protein, Ricin A subunit

## Abstract

Ricin is a potent plant toxin that targets the eukaryotic ribosome by depurinating an adenine from the sarcin-ricin loop (SRL), a highly conserved stem-loop of the rRNA. As a category-B agent for bioterrorism it is a prime target for therapeutic intervention with antibodies and enzyme blocking inhibitors since no effective therapy exists for ricin. Ricin toxin A subunit (RTA) depurinates the SRL by binding to the P-stalk proteins at a remote site. Stimulation of the *N*-glycosidase activity of RTA by the P-stalk proteins has been studied extensively by biochemical methods and by X-ray crystallography. The current understanding of RTA’s depurination mechanism relies exclusively on X-ray structures of the enzyme in the free state and complexed with transition state analogues. To date we have sparse evidence of conformational dynamics and allosteric regulation of RTA activity that can be exploited in the rational design of inhibitors. Thus, our primary goal here is to apply solution NMR techniques to probe the residue specific structural and dynamic coupling active in RTA as a prerequisite to understand the functional implications of an allosteric network. In this report we present *de novo* sequence specific amide and sidechain methyl chemical shift assignments of the 267 residue RTA in the free state and in complex with an 11-residue peptide (P11) representing the identical C-terminal sequence of the ribosomal P-stalk proteins. These assignments will facilitate future studies detailing the propagation of binding induced conformational changes in RTA complexed with inhibitors, antibodies, and biologically relevant targets.

## Biological context

Ribosomes play a central role in cellular survival by translating genetic information to direct the biosynthesis of nascent polypeptide chains in a dynamic macromolecular assembly (Steitz 2008; Korostelev et al. 2008; Noeske and Cate 2012). The translational activity is highly susceptible to a family of lethal toxins known collectively as ribosome-inactivating proteins (RIP), which disable ribosome function and trigger apoptosis. These toxins have evolved in diverse organisms such as ricin in plants, alpha-sarcin in fungi and Shiga toxins I/II in pathogenic bacteria (Barbier and Gillet 2018) and are viewed as important targets for developing antibody (Rudolph et al. 2014) and small molecule inhibitors (Tanaka et al. 2001; Li et al. 2021). The domain structure of ricin includes an enzymatically active A subunit (RTA) and a cell binding lectin B subunit (RTB) linked by a disulfide bond (Rutenber and Robertus 1991). The catalytic domain of RTA is an rRNA *N*-glycosidase which depurinates A4324 in eukaryotic 28 S rRNA or A2660 in *E. coli* 23 S rRNA in the highly conserved sarcin-ricin loop (SRL) by cleaving an *N*-glycosidic bond (Endo and Tsurugi 1987). In the ribosome, the SRL rRNA along with the P-stalk proteins form the GTPase associated center involved in regulating mRNA translation (Steitz 2008; Maracci and Rodnina 2016). The highly conserved tetraloop (GAGA) within the SRL rRNA (Szewczak et al. 1993) is selectively depurinated by the RIP toxins (Amukele and Schramm 2004), which in turn inhibit various translation factor interactions within the ribosome debilitating tRNA-mRNA translocation (Voorhees et al. 2010; Shi et al. 2012; Grela et al. 2019).

In the intact ribosome, RTA is tethered to the eukaryotic P-stalk proteins, which facilitate access to the SRL rRNA substrate for steric reasons (Chiou et al. 2008; Li et al. 2010). The association involves interactions between the identical C-terminal 11 residues of the five P-stalk proteins on the ribosome and the P-stalk binding site located in the C-terminal domain of RTA (Li et al. 2018). Blocking the P-stalk binding site with peptide mimics (Li et al. 2018), small molecules (Li et al. 2021) or antibodies (Czajka et al. 2022) suppresses the enzymatic activity of RTA very efficiently despite being physically remote from the active site. This ability to inhibit depurination indirectly underscores the importance of the P-stalk binding site as a novel target for allosteric inhibitors that can overcome the existing discovery challenges of creating inhibitors targeting the active site of RTA (Jasheway et al. 2011; Li et al. 2020).

To date ricin complexed with various inhibitors and antibodies exhibits limited structural variation contrary to evidence from MD simulations, which suggest that dynamics plays an important role in substrate recognition (Olson 1997) and possibly allosteric inhibition by neutralizing antibodies (Dai et al. 2011). More recently the CryoEM structure of the eukaryotic P-stalk pentamer and Shiga toxin 2a (Stx2a), a close homologue of RTA revealed significant conformational heterogeneity in the C-terminal P-stalk binding site (Kulczyk et al. 2023).

An outstanding question in the structural biology of RTA is the nature of the communication between the active site and the P-stalk binding site separated by more than 20Å. Mutational studies concluded that the ~ 200-fold enhancement in the efficiency of depurination observed in the presence of the pentameric P-stalk proteins cannot be attributed solely to the local concentration effect (Li et al. 2013). The prevailing hypothesis is that binding of different modalities at the P-stalk site or at the N-terminal domain (Dai et al. 2011) of RTA may be communicated to the catalytic site of the enzyme by an unknown allosteric mechanism. A distinctive feature of allosteric regulation is coupled conformational and dynamic changes facilitated by a network of intramolecular interactions. Hence mapping such correlated changes is the first step to gain important mechanistic insight into the regulation of the depurination activity of the RIP toxins and to rationalize the high turnover of RTA.

High resolution solution NMR is a versatile technique that has been widely employed to explore the conformational landscape of enzymes with tunable dynamics while demonstrating the effects of structural allostery at the atomic level (Palmer 2015; Alderson and Kay 2021). Our primary objective here is to obtain backbone amide (NH) and methyl group chemical shift assignments (CH3), which will serve as a foundation for future studies comparing RTA interactions with different inhibitors while investigating the role of dynamics in enzyme function.

## Methods and experiments

### Protein expression

The RTA gene (residues 1-267) from *Ricinus communis* (castor bean) with two mutations (V76M and Y80A) was subcloned into a modified (original His_6_) pET His_10_ Sumo TEV LIC cloning vector (1S) (Addgene plasmid # 29,659). The mutations significantly neutralize the toxin but preserve the basic structure of RTA (Legler et al. 2011). Isotopically labeled protein was expressed in BL21(DE3) pLysS cells. Bacterial cultures were grown in 2 L of LB (OD_600_ = 0.7) before spinning down the cells (1500*g*) and transferring the pellet to warm 30 ml M9 media without carbon/nitrogen source and left to shake for 15–30 min at 37 °C. The cells were spun down (1500*g*) and transferred into warm 500 mL of deuterated (100% D_2_O) or protonated (100% H_2_O) M9 minimal media supplemented with ^15^N-ammonium chloride (1 gm/L), ^13^C-Glucose (4 gm/L), trace elements and antibiotics (Studier 2005). After 1 h at 37 °C, protein expression was induced by 1 mM IPTG, and the cells grown overnight (16 h) at 20 °C (~ 22 h in D_2_O). The labeled protein was purified by a previously described protocol involving nickel affinity column and size exclusion chromatography (Czajka et al. 2022). After removing the N-terminal SUMO-His_10_ tag by TEV cleavage, the purified protein was concentrated and exchanged into NMR buffer with trace protease inhibitors (Roche protease Inhibitor EDTA free tablets), 50 mM Hepes Buffer, 150 mM NaCl, 1 mM TCEP in 95% H_2_O/5% D_2_O at pH 7.5.

### NMR spectroscopy

The NMR data were acquired on Bruker *AVANCE* III spectrometers equipped with TCI CryoProbes™ at 18.8T. Two independent samples of RTA with ^1^H/^13^C/^15^N-labeled (190 µM) and ~ 80% deuterated ^13^C/^15^N-labeled (250 µM) protein were used for sequence specific backbone and side-chain chemical shift assignments. A standard suite of triple-resonance (HNCO, HNCA, HN(CO)CA, HNCACB, HN(CO)CACB) experiments (Sattler et al. 1999), 3D ^15^N-edited NOESY-HSQC and 3D (^15^N,^15^N)-edited HSQC-NOESY-HSQC were acquired on the deuterated U-^13^C/^15^N-labeled RTA (250 µM) at 18.8T. Due to some loss of signal from slow back exchange of amide protons in the beta-sheet of the protein when expressed in D_2_O, additional assignments were obtained from 3D HNCA, HNCO, CBCA(CO)NH, ^15^N-edited NOESY-HSQC and 3D (^13^C^aliphatic^,^15^N^amide^)-edited HSQC-NOESY-HSQC spectra acquired on a uniformly protonated ^13^C/^15^N-labeled RTA (190 µM) sample. The side-chain methyl resonances were assigned using a combination of 3D (H)CCH-COSY, (H)CCH-TOCSY, 3D ^13^C-edited NOESY-HSQC, and 3D (^13^C^aliphatic^,^13^C^methyl^)-edited HMQC-NOESY-HMQC experiments acquired on the protonated sample at 18.8T (Sattler et al. 1999; Cavanagh 2006). The mixing time for the DIPSI2 sequence is set to 16 ms in (H)CCH-TOCSY, and 100 ms in the ^1^H-^1^H NOE experiments.

To map the binding site of the P-stalk peptide (P11) (Li et al. 2018), C^13^/N^15^-labeled RTA (220 µl and 200 µM) was titrated with 5 µl of 10 mM unlabeled P11 peptide (GenScript) dissolved in NMR buffer. The shifts in the amide and methyl peaks were followed during the titration (8 points) by acquiring 2D ^1^H-^15^N HSQC and 2D ^1^H-^13^C HMQC spectra respectively at 18.8T after each peptide addition until saturation is reached at ~ 1:9 protein-to-peptide ratio. In the P11 peptide complex the methyl peaks are in fast exchange between the free and bound state. By following the trajectory of the peaks, we were able to assign all the methyl resonances in the complex. Additionally, the backbone assignments in the peptide complex were confirmed by 3D HNCA, HNCO and 3D N^15^-edited NOESY-HSQC. All the NMR data were processed in Topspin 3.5 from Bruker Biospin and the spectra analyzed in CARA 1.5 (Keller 2004).

## Extent of assignment and data deposition

**Fig. 1:**
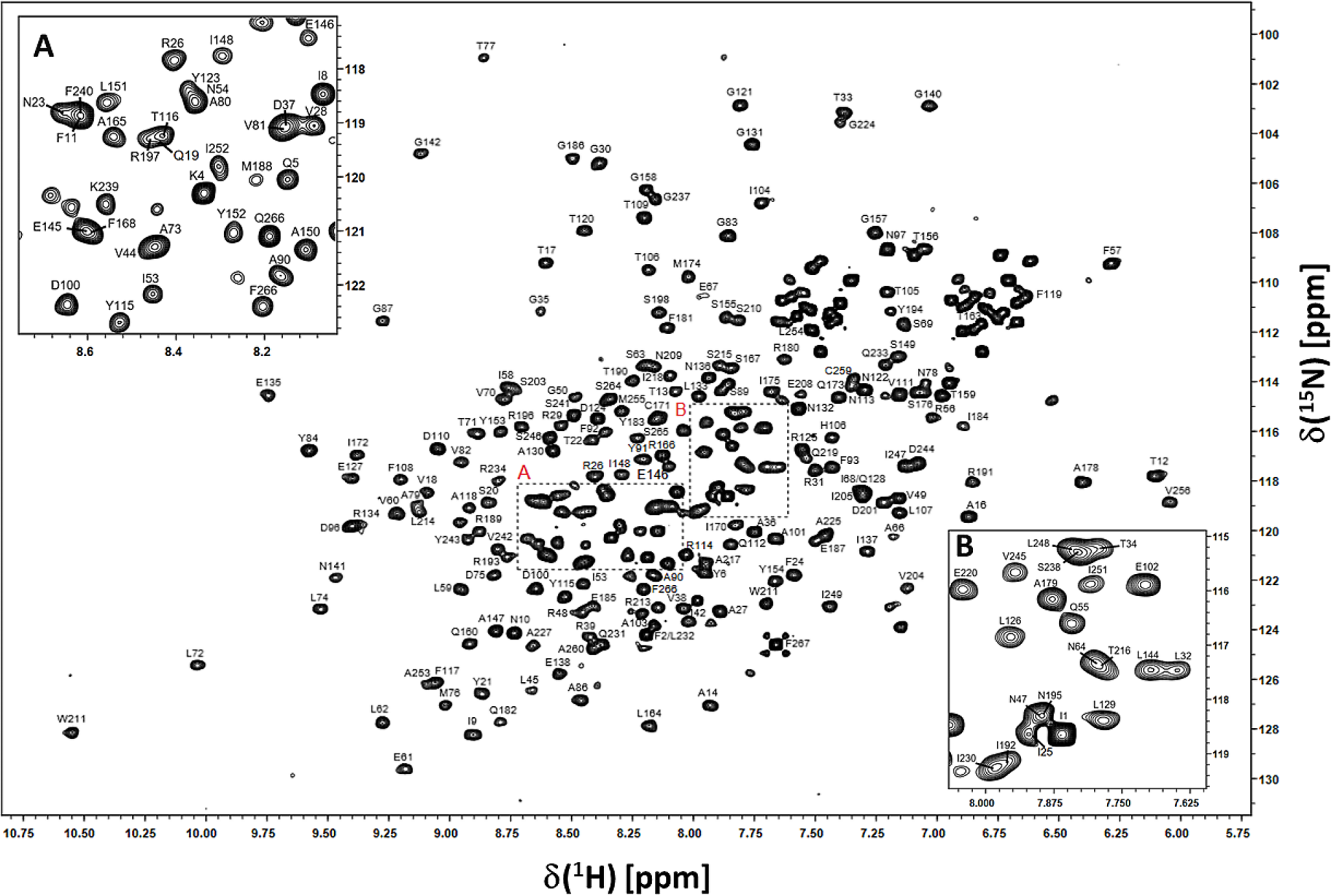
Annotated ^1^H-^15^N HSQC spectrum of 190 µM ^13^C/^15^N labeled RTA (267 residues) in 50 mM Hepes, 1 mM TCEP, 95% H_2_O/5% D_2_O at pH 7.5. The data shown was acquired on 800 MHz Bruker *Avance III* spectrometer at 298°K

Ricin A subunit is a 30 kDa protein composed of 267 residues which includes 15 prolines in the sequence. The excellent dispersion of resonances in the 2D ^1^H-^15^N HSQC spectrum (Fig. 1) allowed us to assign greater than 90% of the amide resonances in the 267-residue protein in the free state and P-stalk peptide complex (Li et al. 2018). Using the backbone walk and ^1^H^N^-^1^H^N^ NOESY correlations we were able to assign all the observed ^1^H-^15^N correlations in the 2D N15-edited HSQC to 235 residues (93% of 252) (Fig. 1). Including the prolines, we have assignments for 97% C^α^ (260/267), 93% C^β^ (233/250) and 88% C’ (234/267) carbon atoms, respectively. The missing and unassigned amide resonances generally belong to residues located in flexible loops or are exchange broadened due to adjacent prolines. Notably the amide resonances of nearly all residues at functionally important sites could be assigned except for Glu177 in the active site.

Methyl groups from hydrophobic sidechains are excellent probes of conformational flexibility in large proteins, and useful for monitoring binding induced structural changes. By combining intra-residue carbon-carbon spin correlations from 3D (H)CCH-TOCSY with through space NOE based connectivities, we were able to assign 93% of 125 methyl groups from Ala (26 C^β^), Ile (22/23 C^δ1^), Leu (37/44 C^δ^^1^ and C^δ^^2^), Met (4 C^ε^) and Val (27/28 C^γ^^1^ and C^γ^^2^).

**Fig. 2:**
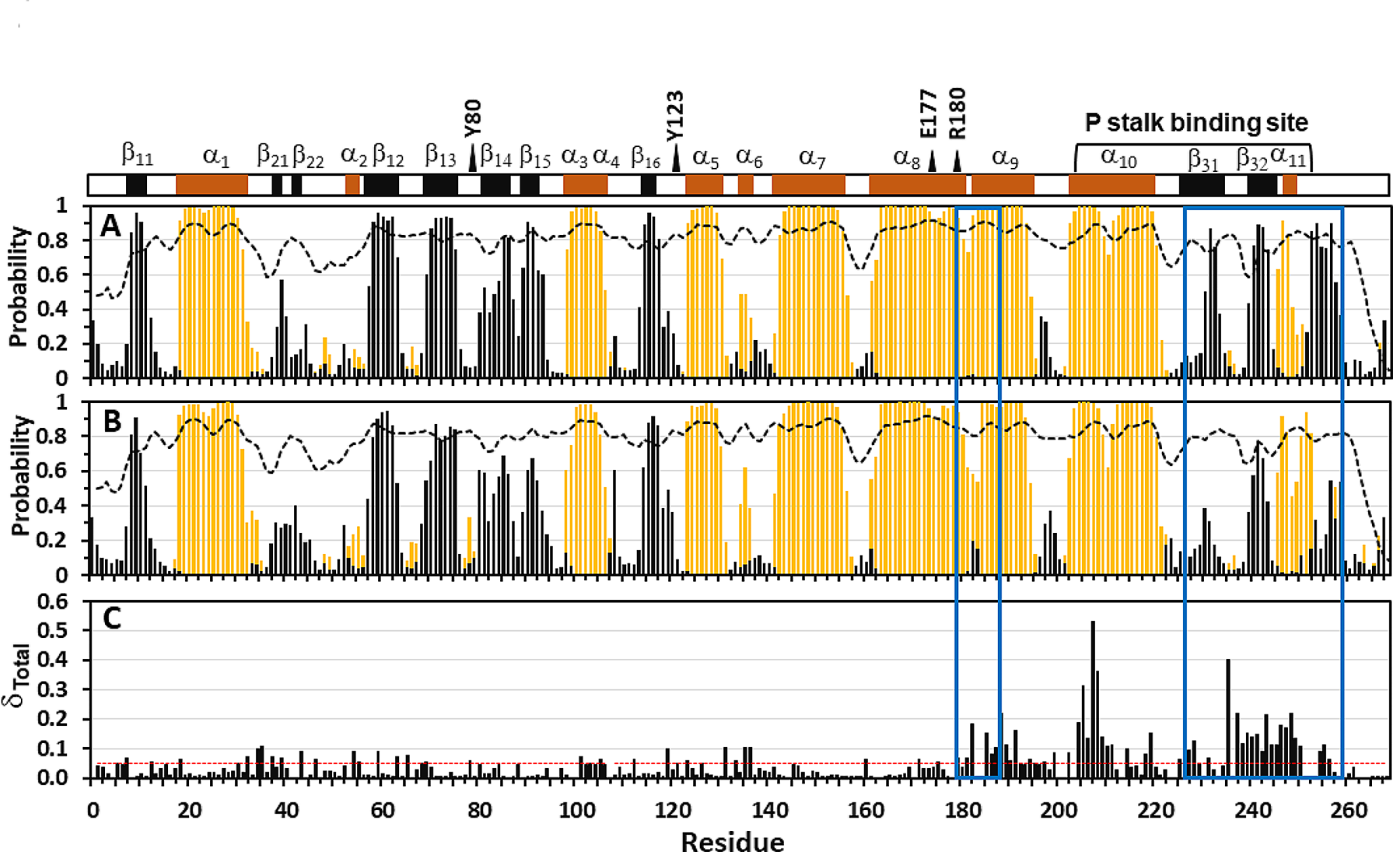
Secondary structure probabilities from TALOS-N analysis of the backbone chemical shifts (N, C’, C^α^, C^β^), (**A**) RTA, and (**B**) RTA in complex with P11 peptide. In the bar plot the helical propensity is indicated in yellow and strands in black. The blue boxes highlight correlated changes in coupled sites. The active site residues and the secondary structure defined in the X-ray structure (PDB code 1RTC) is displayed in the top panel. The measure of backbone disorder (0 < S^2^ < 1) in the unstructured regions is represented by the random coil index values (RCI-S^2^) plotted as dotted line. (**C**) Histogram of the weighted average of the total chemical shift difference between free protein and RTA bound to P11 peptide, calculated using the relationship √[(Δδ_H__N_)^2^ + (0.154*Δδ_N_)^2^ + (0.276*Δδ_C__α_)^2^] (Evenas et al. 2001). The minimum threshold of chemical shift perturbation (~ 0.05 ppm) is indicated by the dotted red line

**Fig. 3:**
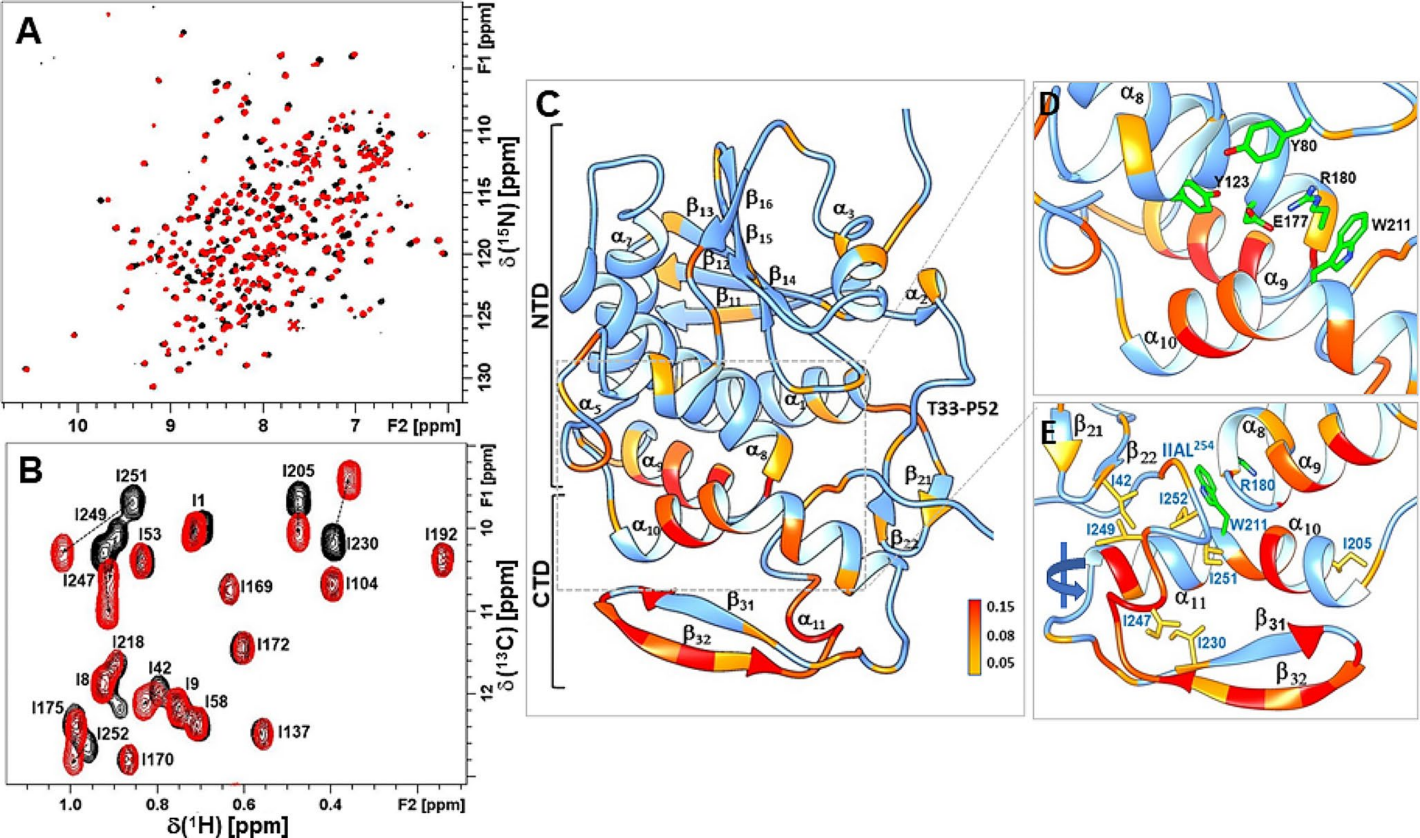
Overlay of 800 MHz spectra of ^13^C/^15^N labeled RTA (black) complexed with P11 peptide (red) at 298 K. (**A**) ^1^H-^15^N HSQCs, (**B**) ^1^H-^13^C HMQCs showing the selective perturbation of ILE C^δ1^H_3_-methyl cross-peaks at the P-stalk binding site in the P11 peptide bound RTA complex. (**C**) The weighted average of the total chemical shift difference (Fig. 2c) was mapped onto the RTA structure (PDB 1RTC) in Chimera 1.16 (Pettersen et al. 2004). The magnitude of perturbation (ppm) is color coded as per legend. Displayed insets, (**D**) Active site residues, and (**E**) Isoleucines in the P-stalk binding site (180° rotation) shown in stick representation

The backbone (N, C’, C^α^, C^β^) chemical shifts were analyzed using TALOS-N (Shen and Bax 2015) and the secondary structure predictions compared to the X-ray structure of RTA (Mlsna et al. 1993) (Fig. 2a). The random coil index order parameter (RCI-S^2^) is a useful parameter to identify relatively dynamic regions of the backbone with values ranging between S^2^ = 0 (disordered) to S^2^ = 1 (rigid structure). The TALOS secondary structure predictions are broadly consistent with the αβ-fold present in the N-terminal domain (residues 1-200, NTD) linked to the smaller C-terminal domain (CTD) where the P stalk binding site is located. The opening between the two domains encloses the active site of the enzyme lined by residues important for docking the SRL rRNA sequence (Marsden et al. 2004). Compared to the well-defined core of the protein, the most variable region (0.6 < RCI-S^2^ < 0.8) is the unstructured loop linking α_1_ and β_12_ (residues 33 to 52). The tip of the loop extends across the active site and makes contacts with both α_1__0_ at the P-stalk binding site and CTD (Fig. 3c). Notably the P-stalk binding site scaffold is not as well defined as observed in the X-ray structures with increased backbone variation in β_31_ compared to the more stable β_32_ strand. The remaining C-terminal backbone is essentially random coil, with the notable exception of residues Ile251 to Leu254 which adopts a strand-like extended conformation (~ 80%) stabilized by interactions with the hinge (Phe181 - Tyr183) flanked by α_8_ and α_9_ helices.

To probe the extent of binding induced conformational changes in RTA triggered by the P-stalk peptide, amide (Fig. 3a) and methyl (Fig. 3b) resonances of the protein in complex with the P11 peptide were reassigned. The results from the TALOS analysis of the backbone chemical shifts (N, C^α^, C') is plotted in Fig. 2b. The CSP profile (Fig. 2c) is mapped on the three-dimensional structure (Fig. 3c). Some of the largest chemical shift perturbations are clustered in the C-terminal domain which can be rationalized by significant structural rearrangement at the intermolecular contact surface in the complex. These changes are relayed to the N-terminal domain where long-range effects are observed in α_1_, α_3_, α_8_-α_10_ and loops within the active site (Tyr80, Tyr123, Glu177 and Arg180) (Fig. 3d) and the nucleotide binding site (Marsden et al. 2004).

The remodeling of the CTD is well supported by the TALOS results (Fig. 2a and b), which reflect a shift in the secondary structure preferences at the P-stalk site without concomitant changes in the RCI-S^2^ profiles. In the NTD, localized changes are detected in the Thr33 - Pro52 loop and β_14_-β_15_ strands. A notable dip in RCI-S^2^ values in the α_8_-α_9_ hinge region (Phe181 - Tyr183) can be directly linked to the interaction (Fig. 3e) with the C-terminal residues ^251^IIAL^254^ reconfigured in the complex (Fig. 2b). The proximity of the active site (Glu177 and Arg180) to the α_8_-α_9_ hinge residues (Fig. 3d), suggests this interaction with the pliable C-terminal domain could be mechanistically important and warrants further investigation.

To summarize the conclusions, we have achieved near complete backbone and side-chain methyl assignments of RTA in the presence and absence of the P-stalk peptide. The secondary structure analysis and CSP mapping presents clear evidence of coupling between the P-stalk site and the active site. The data indicates that the core domain structure is unaffected by the P-stalk interaction, but the selective rearrangement of a subset of residues agrees with the presence of an allosteric network which mediates communication between physically distant sites (Gianni and Jemth 2023). These initial results will be the cornerstone of further studies characterizing the site-specific structural and dynamic response of various ligands with the goal of understanding the functional role of conformational flexibility in allosteric regulation of the enzyme.

## Data Availability

The plasmids used for producing the protein are available upon reasonable request from the authors and the chemical shifts have been deposited with the BioMagResBank (https://bmrb.io/) under entry number 52271.
